# Supplementary material for: Air Pollution Exposure Monitoring among Pregnant Women with and without Asthma
Source: Int J Environ Res Public Health. 2020 Jul 7;17(13):4888. doi: 10.3390/ijerph17134888 (PMC7369909; doi:10.3390/ijerph17134888)
Supplement: Supplementary file 1 [file ijerph-17-04888-s001.pdf]

Air pollution exposure monitoring among pregnant women with and without asthma

Sandie Ha[1], Carrie Nobles[2], Jenna Kanner[2], Seth Sherman[3], Seung-Hyun Cho[4], Neil Perkins[2], Andrew Williams[5], William Grobman[6], Joseph Biggio[7], Akila Subramaniam[8], Marion Ouidir[2], Zhen Chen[2], Pauline Mendola[2]

Affiliations:

[1]Department of Public Health, College of Social Sciences, Humanities, and Arts, Health Sciences Research Institute, University of California, Merced, CA, USA

[2]Division of Intramural Population Health Research, *Eunice Kennedy Shriver* National Institute of Child Health and Human Development, Bethesda, MD, USA

[3]The Emmes Company, Rockville MD, USA

[4]RTI International, Research Triangle Park, NC, USA

[5]School of Medicine & Health Sciences, University of North Dakota, Grand Forks, ND, USA

[6] Feinberg School of Medicine, Northwestern University, Chicago IL, USA

[7]Ochsner Health System, New Orleans LA, USA

[8]The University of Alabama at Birmingham, Birmingham AL, USA

Supplemental Figures and Tables	Page
Figure S1. Wearable sensors used in the study: RTI MicroPEM, Cairpol CairClip, and Ogawa O_3 passive sampler	2
Figure S2. The air monitoring sensors in the monitoring platform worn by a model pregnant mother, mesh pouch including all monitors, and shipping tray	2
Figure S3. Distribution of air pollution exposure by asthma status	3
Table S1. Selected characteristics of study areas	4
Table S2. Training video clips for air monitoring step-by-step instructions	5
Table S3. Air pollution distributions by asthma status for Site 1.	6
Table S4. Air pollution distribution by study site	7
Table S5. Correlation matrix for different pollutants across assessment methods.	8

Figure S1. Wearable sensors used in the study: RTI MicroPEM (left), Cairpol CairClip (middle), and Ogawa O_3 passive sampler (right)

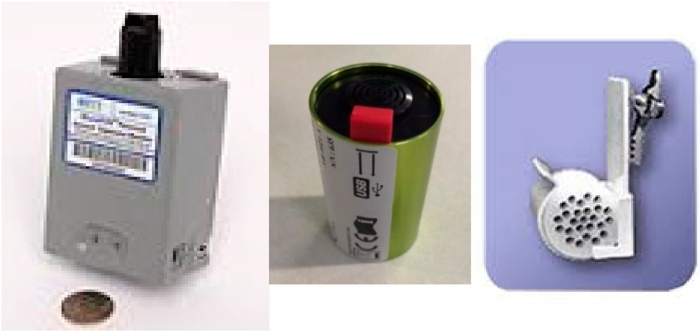

Figure S2. The air monitoring sensors in the monitoring platform worn by a model pregnant mother (left), mesh pouch including all monitors (middle), and shipping tray (right).

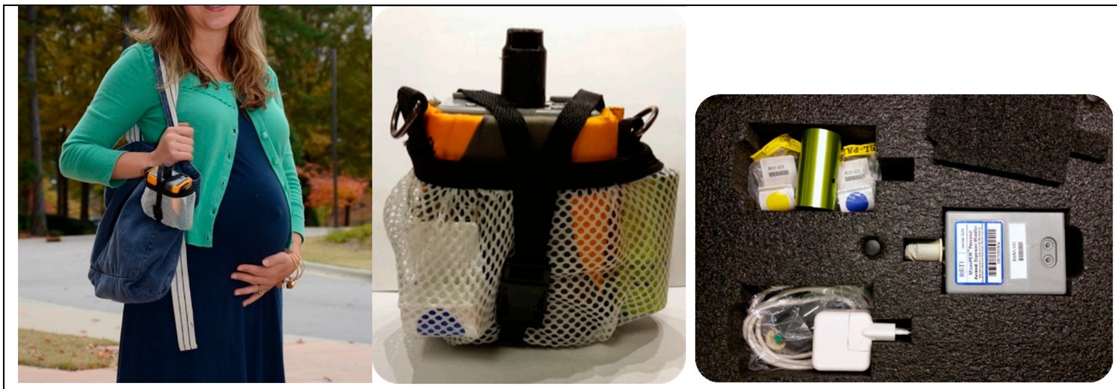

Figure S3. Distribution of air pollution exposure by asthma status.

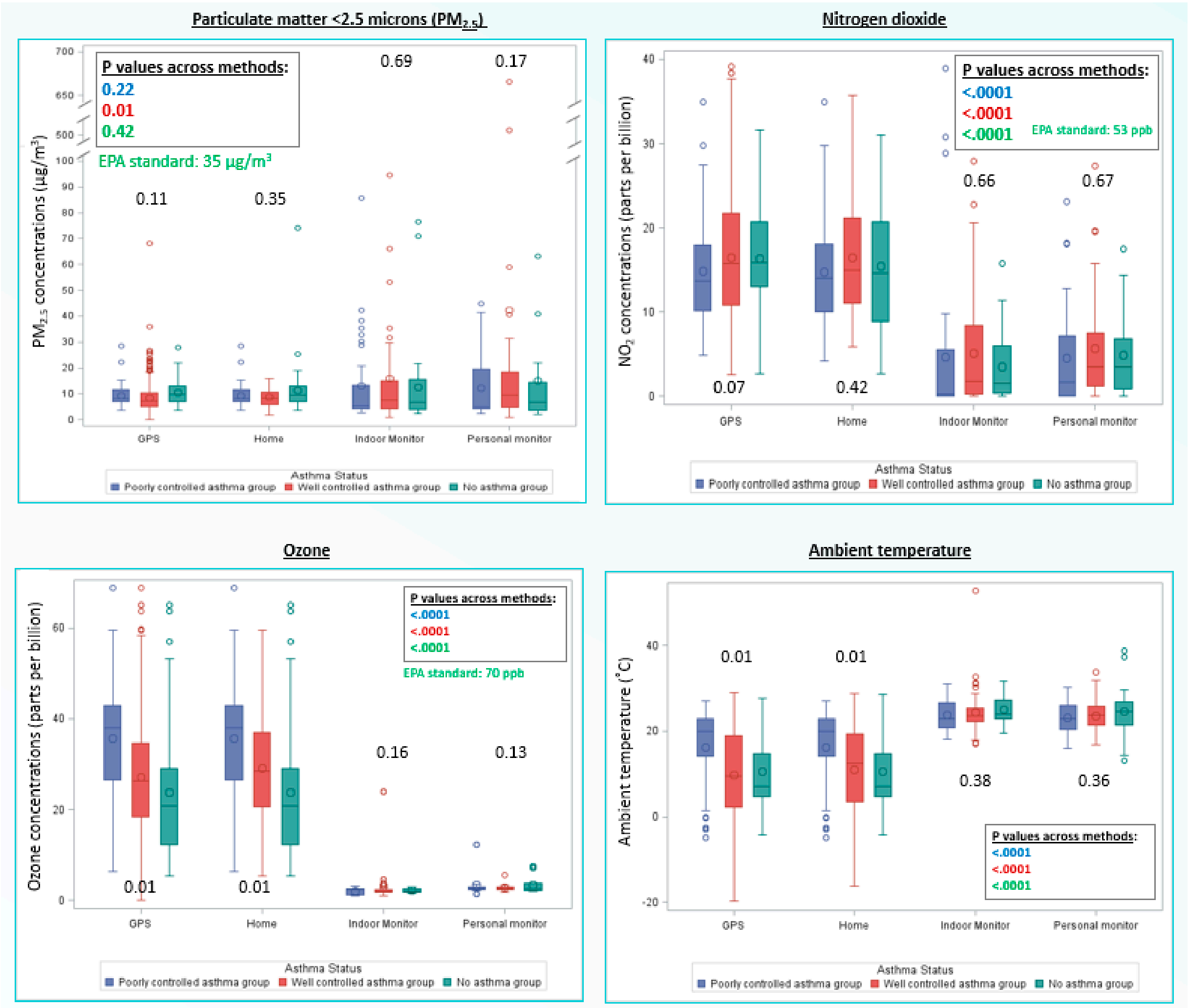

*P-values were obtained from mixed models to account for within person autocorrelation.

Table S1. Selected characteristics of study areas

Characteristics	Chicago, IL	Birmingham, AL
Population size (Census 2010)	2,722,586	212,265
Female Population	51.50%	52.70%
Median Age	34.1	35.7
Race/ethnicity		
White	32.70%	22.80%
Black	30.10%	71.30%
Asian	6.20%	0.80%
Native American	0.10%	0.20%
Hawaiian, Pacific Islander	0.00%	0.00%
Other	0.20%	0.20%
Two or More Races	1.70%	1.20%
Hispanic	29.00%	3.50%
Air pollution (2015-2018) (EPA AirData)		
NO_2 (ppb)	51.3(14.2)	40.2(15.9)
Ozone (ppb)	70.9(31.9)	55.6(23.9)
$PM_{2.5}$ (ug/m3)	56.8(16.8)	51.7(13.8)
Annual temperature (US Climate Data)		
Average low	5.5 ˚C (42 F)	11.7 ˚C (53 F)
Average high	13.9 ˚C (57 F)	23.3 ˚C (74 F)

Table S2. Training video clips for air monitoring step-by-step instructions

Video Clip Descriptions	YouTube Link
Box Receipt at Clinic	http://youtu.be/LjlBUvTSg8I
Load MicroPEM™ battery	http://youtu.be/D-e3JC4svlg
Start MicroPEM	http://youtu.be/iU4uyPjcdL8
Start CairClip	http://youtu.be/_6DaVSymHpg
Start Ogawa	http://youtu.be/BiuRjT3G8AI
How to Wear Personal Monitors	http://youtu.be/1QO83shPRMQ
Setting Up Indoor Monitors	http://youtu.be/R3ACi_8LNR4
End Monitoring and Pack Box	http://youtu.be/Gv02ZfhDvQw

Table S3. Air pollution distributions by asthma status for Site 1.

Pollutant	Assessment method[a]				
		No asthma	Well controlled	Poorly controlled	p-value[b]
$PM_{2.5}$ (µg/m^3)	Home + EPA monitor	9.6(3.6-19)	8.5(1.7-16)	9.2(3.6-28.5)	0.4399
	GPS + EPA monitor	10.2(3.6-28)	8.4(1.7-14.6)	9.2(3.6-28.5)	0.1326
	Indoor	11.8(2.3-76.5)	15.6(1-132.8)	10(2.5-42.2)	0.4283
	Personal	10.9(2.1-63.2)	43.2(0.9-665.6)	10.4(2.4-44.7)	0.1545
	p-value exposure[c]	0.8825	0.0120	0.8627	
Ozone (ppb)	Home + EPA monitor	23.5(5.3-65)	28.4(5.4-59.5)	33.9(6.3-68.8)	0.0028
	GPS + EPA monitor	23.5(5.3-65)	28.5(5.4-59.5)	33.9(6.3-68.8)	0.0028
	Indoor	2.1(1.6-2.7)	3.3(1.1-23.9)	2(1.1-2.8)	0.1242
	Personal	3.5(1.9-7.6)	2.8(1.9-5.5)	3.6(1.5-12.2)	0.1343
	p-value exposure[c]	<.0001	<.0001	<.0001	
NO_2 (ppb)	Home + EPA monitor	17.4(6.9-31)	15.7(5.9-35.8)	16.5(4-35)	0.3908
	GPS + EPA monitor	18.5(7.9-31.6)	15.6(5.5-34.7)	16.4(4-35)	0.0541
	Indoor	3.7(0-15.8)	5.4(0-27.9)	5.1(0-39)	0.6489
	Personal	5.1(0-17.4)	5.8(0-27.4)	4.6(0-23.1)	0.6724
	p-value exposure[c]	<.0001	<.0001	<.0001	
Temperature (◦C)	Home + EPA monitor	8(-4.4-28.5)	12.1(-16.3-28.8)	15.9(-4.8-27)	0.0012
	GPS + EPA monitor	8(-4.4-27.8)	12(-16.3-28.8)	15.9(-4.8-27)	0.0011
	Indoor	24.9(19.5-31.7)	24(17.1-52.8)	23(18.1-28.3)	0.1673
	Personal	23.9(13.1-38.7)	23.1(16.8-27.6)	22.3(16-28)	0.2773
	p-value exposure[c]	<.0001	<.0001	0.0002	

Abbreviation: GPS, global positioning system; EPA, US Environmental Protection Agency; $PM_{2.5}$, particulate matter <2.5 microns; NO_2, nitrogen dioxide

[a]The GPS and home method includes 24 participants, the personal monitoring method analysis includes 39 participants, and the indoors method include 40 participants.

[b]p-values were obtained cross asthma status by mixed models to account for within person variation.

[c]p-values were obtained cross assessment methods by mixed models to account for within person variation

Table S4. Air pollution distribution by study site

Pollutant	Assessment method[a]	Mean (min-max)		
		NWU	UAB	p-value[b]
$PM_{2.5}$ (µg/m^3)	Home + EPA monitor	9(1.7-28.5)	10.6(5.8-13.3)	0.4088
	GPS + EPA monitor	9.2(1.7-28.5)	10.6(5.8-13.3)	0.5066
	Indoor	13.2(1-132.8)	23.2(4-85.8)	0.1114
	Personal	26.5(0.9-665.6)	28.6(4.1-122.3)	0.9313
	p-value exposure[c]	0.0040	0.4803	
Ozone (ppb)	Home + EPA monitor	28.7(5.3-68.8)	34.6(25.7-42.2)	0.3247
	GPS + EPA monitor	28.8(5.3-68.8)	34.6(25.7-42.2)	0.3264
	Indoor	2.7(1.1-23.9)	2.2(1.2-3.2)	0.5882
	Personal	3.2(1.5-12.2)	2.7(2.1-3.7)	0.3714
	p-value exposure[c]	<.0001	0.0005	
NO_2 (ppb)	Home + EPA monitor	16.3(4-35.8)	16.1(10.4-20)	0.9440
	GPS + EPA monitor	16.5(4-35)	16.1(10.4-20)	0.8873
	Indoor	5(0-39)	0.5(0-2.1)	0.0689
	Personal	5.3(0-27.4)	2.9(0.1-8.1)	0.2391
	p-value exposure[c]	<.0001	0.0165	
Temperature (◦C)	Home + EPA monitor	12.3(-16.3-28.8)	26(24.7-27)	0.0043
	GPS + EPA monitor	12.3(-16.3-28.8)	26.1(24.8-27.2)	0.0040
	Indoor	23.9(17.1-52.8)	28(21.8-31.3)	0.0007
	Personal	23.1(13.1-38.7)	28.8(24.5-37.3)	<.0001
	p-value exposure[c]	<.0001	0.3580	

Abbreviation: GPS, global positioning system; EPA, US Environmental Protection Agency; $PM_{2.5}$, particulate matter <2.5 microns; NO_2, nitrogen dioxide

[a]The GPS and home method includes 24 participants, the personal monitoring method analysis includes 39 participants, and the indoors method include 40 participants.

[b]p-values were obtained cross sites by mixed models to account for within person variation.

[c]p-values were obtained cross assessment methods by mixed models to account for within person variation

Table S5. Correlation matrix for different pollutants across assessment methods.

Pollutant	Assessment method	Personal	Indoor	GPS+EPA monitor	Home+EPA monitor
$PM_{2.5}$[a]	Personal	1.00	0.32	-0.21	-0.21
	Indoor	0.32	1.00	-0.21	-0.19
	GPS+EPA monitor	-0.21	-0.21	1.00	0.99
	Home+EPA monitor	-0.21	-0.19	0.99	1.00
Ozone[b]	Personal	1.00	-0.04	0.45	0.45
	Indoor	-0.04	1.00	-0.13	-0.12
	GPS+EPA monitor	0.45	-0.13	1.00	1.00
	Home+EPA monitor	0.45	-0.12	1.00	1.00
NO_2	Personal	1.00	0.76	-0.27	-0.25
	Indoor	0.76	1.00	-0.32	-0.23
	GPS+EPA monitor	-0.27	-0.32	1.00	0.96
	Home+EPA monitor	-0.25	-0.23	0.96	1.00
Temperature	Personal	1.00	0.76	0.56	0.57
	Indoor	0.76	1.00	0.62	0.62
	GPS+EPA monitor	0.56	0.62	1.00	1.00
	Home+EPA monitor	0.57	0.62	1.00	1.00

[a] indoor and personal estimates are assessed sequentially, and do not reflect the same dates.

[b] Ozone was measured as an aggregate estimate over the entire study period
